# Multisystem inflammatory syndrome (MIS-C) with SARS-CoV-2 omicron variant BA.2.38 in a four-year-old Chinese girl: A case report

**DOI:** 10.3389/fpubh.2022.1021200

**Published:** 2022-11-09

**Authors:** Wen-yuan Wang, Yong-jun Wang, Cai-xia An, Qi-jun Zhao, Shu-ying Wang, Wan-yi Li, Bin Yi, Huan Li

**Affiliations:** ^1^Pediatric Respiratory Department II, Gansu Provincial Maternity and Child-Care Hospital, Lanzhou, China; ^2^Department of Rehabilitation, Gansu Province Hospital Rehabilitation Center, Lanzhou, China

**Keywords:** SARS-CoV-2, multisystem inflammatory syndrome, MIS-C, COVID-19, children

## Abstract

We report a severe COVID-19 complicated with MIS-C in a girl treated by the author in China, and discuss the current research status and progress in the diagnosis and therapy of MIS-C in children. The patient was a 4-year-old child previously healthy who was referred to the hospital with a complaint of fever, finally, Multisystem inflammatory syndrome was diagnosed with COVID-19.

Since the outbreak of coronavirus disease 2019 (COVID-19), severe cases in children in China have been significantly lower than in adults. In particular, Multisystem inflammatory syndrome in children (MIS-C) is scarcely reported in China. In late April 2020, clinicians in the United Kingdom reported a cluster of eight previously healthy children presenting with hyperinflammatory shock during the COVID-19 pandemic, showing features similar to atypical Kawasaki disease (KD), Kawasaki disease shock syndrome, or toxic shock syndrome (1). The Centers for Disease Control and Prevention (CDC) and the World Health Organization (WHO) named the disease multisystem inflammatory syndrome in children (MIS-C). Since the first case was reported, there have been additional similar reports from other countries, primarily in United States, Italy, United Kingdom, France, and Switzerland, and these regions have experienced a considerable number of cases of MIS-C during the SARS-CoV-2 pandemic (1–5). MIS-C has rarely been reported occurring in China in published reports, although COVID-19 was first reported in Wuhan, the worst affected city by SARS-CoV-2 in China. Reasons for this observation are unclear, may involve differences in prevalence rates of infection in children, the infection rate and fatality rate were much lower in China than that of the main European and American epidemic cities; In addition, the differences in ethnic or genetic background and SARS-CoV-2 subtypes, host factors, early large-scale screening and early treatment with immunomodulators may also involve (6). In this article, we report an MIS-C in a girl in China and discuss the current and new progress in the diagnosis and treatment of MIS-C in children based on the literature.

## Case presentation

**Figure 1:**
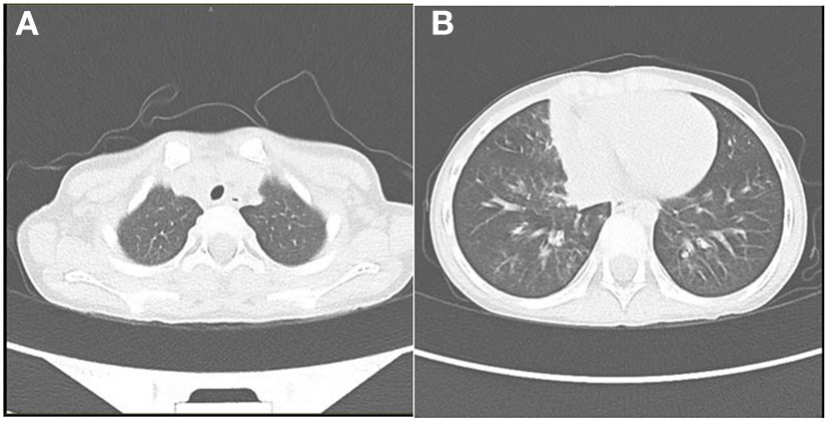
Patient's chest CT showed a few inflammatory lesions in the right upper lobe **(A)**, the middle lobe, and the lower lobe of both lungs and the lower lobe of both lungs **(B)** on the first day of admission.

A 4-year-old female presented to the hospital with a fever for 5 days and was admitted on July 31, 2022. COVID-19 was confirmed by the detection of two SARS-CoV-2 viruses using a nucleic acid test (throat swabs), and the patient was transferred to a designated hospital for treatment. She had a paroxysmal dry cough without obvious dyspnea and continuous fever of up to 41 °C, but without chills or convulsions. She had no gastrointestinal symptoms such as nausea, vomiting, or diarrhea, but with bad appetite. She was previously healthy. Her peripheral oxygen saturation (SpO2) was 78% and SpO2 was 95% under nasal tube oxygen (2 L/min). Temperature(T): 39. 2 °C; pulse (P): 94 times/min; respiration(R): 20 times/min; capillary refill time (CRT): 3s. She had poor mental health, cyanosis of the lips and fingernails, low temperature of the extremities, the flowering of the skin during high fever, congestive rash scattered throughout the body, mild bulbar conjunctival congestion, chapped lips, and a strawberry tongue. Lymph nodes with a diameter of 2 cm were palpable in her right neck, with a range of motion and no tenderness. The liver was palpable 3 cm below the ribs with clear margins, and the spleen below the ribs was not palpable. Laboratory biomarkers (Table 1) indicating inflammation: an elevated white blood cell, erythrocyte sedimentation rate, C-reactive protein level, procalcitonin,interleukin-6; Serum amyloid A, lymphocytopenia, neutrophilia, elevated ferritin level, lactic dehydrogenase, creatine kinase, hypoalbuminemia, and an elevated d-dimer level and fibrinogen level. brain natriuretic peptide (BNP)100pg/ml; troponin 0.004ng/ml.Chest CT showed a few inflammatory lesions in the posterior segment of the right upper lobe, the medial segment of the middle lobe, and the basal segment of the lower lobe of both lungs (Figure 1). Abdominal CT an enlarged liver volume, and echocardiogram demonstrated tricuspid regurgitation rate was 223 cm/s, left ventricular ejection fraction was 65%, and the inner diameters of each heart cavity and great vessels were normal. According to the diagnostic criteria of Diagnosis, treatment, and prevention of severe acute respiratory syndrome coronavirus 2 infections in children: expert' consensus statement (Fourth Edition) (7), She was diagnosed with COVID-19 (severe) with the MIS-C. In our case, the diagnosis was based on the following: (1) serious illness leading to hospitalization; (2) a 4-year-old child with evidence of COVID-19 infection; (3) persistent high fever that lasted for more than 5 days; (4) multisystem organ involvement: respiratory symptoms such as paroxysmal cough; acute gastrointestinal problems such as vomiting and bad appetite; conjunctival hyperemia of both eyes; systemic pleomorphic derma; headache; abnormal coagulation function; (5) evidence of the abnormal elevation of laboratory inflammatory markers that could not be explained by other pathogenic microorganisms.

**Table 1:** Laboratory indexes.

**Measure**	**Day 1**	**Day 3**	**Day 4**	**Day 5**	**Day 6**	**Day 7**	**Reference ranges**
**Complete blood count**	
WBC( × 10^9^/L)	12.2	5.5	4.4	5.3	7.4	5.8	4.4–11.9
HGB (g/L)	119	119	127	119	116	115	112–149
PLT ( × 10^9^/L)	224	209	237	195	190	219	100–300
NEU (%)	88	83	61	77	59	67	22–65
LYM (%)	8.6	14.5	32.7	20.2	32.5	26.9	23–69
MON (%)	3	2	6	3	8	6	2–21
**Inflammatory markers**	
PCT (ng/ml)	5.941	5.242	2.611	1.706	16.84	0.143	0–0.5
CRP (mg/L)	90.08	94.73	58.69	23.65	12.39	7.05	0–10
IL-6 (pg/ml)	96.19	16.9	6.03	3.7	0.59	5.49	1.18–5.30
SAA (ug/ml)	243.6	68.41	41.48	10.09	10.18	21.61	0–10
ESR (mm/h)	-	27	34	-	45	17	0–20
FER (ng/ml)	-	-	170.8	-	-	240.5	25–200
**Coagulopathy**	
PT (s)	13.5	12.1	10.8	9.6	9.5	9.3	9.8–12.1
APTT (s)	38.2	30.6	32	30.4	30.9	26.9	23.3–31.3
INR	1.15	1.03	0.91	0.81	0.8	0.78	0.8–1.5
D-D (ug/ml)	6.89	4.61	1.43	0.82	0.89	1.00	0–0.55
FIB (g/L)	3.64	3.41	3.2	2.24	2.21	2.27	1.8–3.5
**Nucleic acid test results (Omicron BA.2.38)**	
ORF1abgene	34	negative	32	-	negative	negative	negative
N gene	34	37	31	-	negative	negative	negative
IgMDL (S/CO)	-	1.01	-	-	-	1.2	negative
IgGDL (S/CO)	-	29.86	-	-	-	123.65	negative
**Biochemical indicators**
ALB (g/L)	35.8	33.8	34.5	31.7	31.9	33	39–54
GLO (g/L)	21.4	20.7	35.9	42	37.1	49.7	15–34
ALT (U/L)	10	26	24	20	21	24	7–30
AST (U/L)	37	63	43	34	39	31	14–44
TB (umol/L)	13.7	9.9	7.4	5	5.4	3.3	0.14–9.66
GLU (mmol/L)	4.34	3.78	6.6	6.71	4.25	5.7	3.3–5.6
Na+ (mmol/L)	133	133	134.6	134.9	138.4	138.9	135–145
K+ (mmol/L)	3.46	4.46	4.07	3.9	3.36	4.28	3.7–5.2
Ca2+ (mmol/L)	2.1	2.15	2.17	2.14	2.14	2.34	2.5–3.0
LDH (U/L)	448	511	510	415	405	333	120–250
CK (U/L)	149	137	352	283	195	43	40–200
CK-MB (U/L)	16	15	27	44	49	30	0–25
CREA (umol/L)	28	212	27	25	27	22	19–44
UREA(mmol/L)	3.2	5.1	2.8	2.5	2.6	2	2.5–6.5
TG(mmol/L)	0.75	1.61	1.29	1.31	1.47	1.06	0–1.6

“-” indicates undetermined.WBC, white blood cell; HGB, hemoglobin; PLT, platelet count;NEU, Neutrophil; LYM, lymphocytes; PCT, procalcitonin;CRP, C-reactive protein; IL-6, interleukin-6; SAA, Serum amyloid A; ESR, erythrocyte sedimentation rate; FER, ferritin; PT, prothrombin time;APTT, activated partial thromboplastin time; INR, international normalized ratio; D-D, D-dimer;FIB, fibrinogen; ALB, serum albumin; GLO, seroglobulin; ALT, alanine aminotransferase; AST, aspartate aminotransferase; TB, total bile acid; GLU, glucose; LDH, lactic dehydrogenase; CK, creatine kinase;CREA, Creatinine; UREA, urea nitrogen; TG, triglyceride.

**Figure 2:**
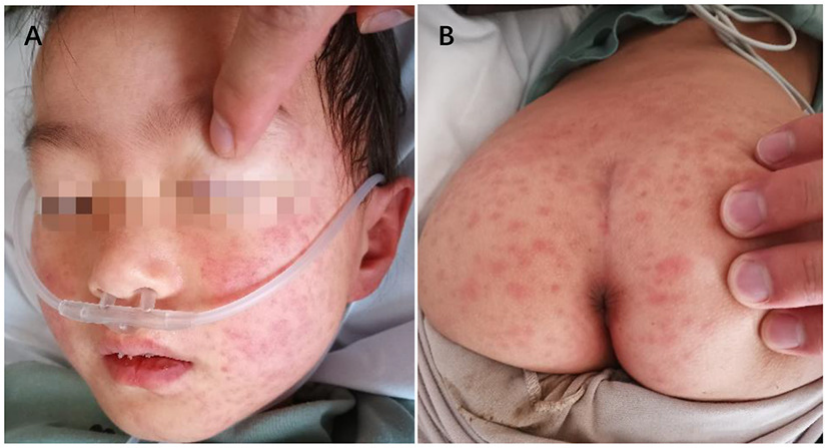
Pleomorphic rash of face **(A)** and trunk **(B)**.

According to Diagnosis, treatment and prevention of severe acute respiratory syndrome coronavirus 2 infection in children: expert' consensus statement (Fourth Edition) (7), the therapy included intravenous immunoglobulin 1 g/kg, once a day for 2 consecutive days, methylprednisolone 1 mg/kg, twice a day for 4 consecutive days, low molecular weight heparin calcium 100 IU/kg subcutaneous injection, twice a day, oral enteric aspirin 5 mg/kg, cough medicine and symptomatic and supportive treatment. On the 4th day, the child developed vomiting and headache for 2 days. On the 6th day after treatment, the patient's temperature gradually returned to normal, and the pleomorphic rash on her face and trunk gradually converged into flakes (Figure 2). The cough disappeared on the 9th day, and the general condition also improved. Most importantly, her SARS-CoV-2 viral nucleic acid test also turned negative on the 9th day, and other laboratory biomarkers indicating inflammation were back to normal. The children were followed up for 2-3 weeks after discharge by gradually reducing the dose of oral methylprednisolone. The specific laboratory results for the children are presented in Table 1. Day 1 is the first day of admission.

## Discussion

We report a previously healthy child who was diagnosed with MIS-C and the clinical manifestations are similar to those of KD, who was improved with intravenous immunoglobulin and systemic glucocorticoids, oral enteric aspirin, subcutaneous injection of low molecular weight heparin based on the elevated d-dimer level.

Severe children cases with COVID-19 are more prevalent in children under 1 year of age, in particular those who utilize immunosuppression, and those with underlying illnesses, and most of them are accompanied by respiratory symptoms, whereas the symptoms are milder than those observed in adults (8). Fever is the main symptom in 93.3% in China, with an average duration of 2–3 days, and most of them have a favorable prognosis. MIS-C is defined as clinically severe illness requiring hospitalization with fever, inflammatory marker elevation, and multisystem organ dysfunction in the setting of recent proven or probable SARS–CoV-2 infection, and in the absence of an alternative likely explanation (9). MIS-C is a severe inflammatory syndrome diagnosed as KD manifested by toxic shock and cardiogenic or vascular paralytic shock. Some children are directly or indirectly related to SARS-CoV-2. It has been reported that MIS-C predominantly occurs in previously healthy children and adolescents (10), in which they are mainly systemic multisystem damage. Patients with MIS-C were noted to have a high frequency of fever and gastrointestinal symptoms including abdominal pain, vomiting, and diarrhea. Cough and respiratory distress were reported not very common or not severe (11). Blood parameters showed neutrophilia in 83% of cases and a high CRP in 94%. However, only 41% demonstrated pulmonary changes on chest imaging. MIS-C is a serious disease that can progress rapidly and worsen in a short time. The severity of illness was high with 68% of cases requiring intensive care admission, 63% requiring inotropic support, 28% of cases needing some form of respiratory support (12), and approximately 60% of patients that were diagnosed with MIS-C presented with shock (11). Studies have reported that the fatality rate of MIS-C in the United Kingdom and the United States can reach 2% (4, 5), which is 10 times higher than the crude fatality rate of about 0.2% for children with COVID-19 reported in the domestic literature (13). At present, MIS-C is scarcely reported from China, which may benefit from our country's epidemic prevention and control policy of “external input and internal rebound”.

### Pathogenesis

SARS-CoV-2 infection is typically very mild and often asymptomatic in children. A complication is the rare MIS-C associated with COVID-19, presenting 4–6 weeks after infection as high fever, organ dysfunction, and strongly elevated markers of inflammation (14). Understanding the pathogenesis of MIS-C will be necessary to inform clinical management and prevention efforts. The underlying pathogenesis has not yet been fully elucidated, while an abnormal immune response is blamed as the main factor in the pathogenesis of MIS-C. Virus-induced post-infective immune dysregulation appears to play a predominant role, with MIS-C commonly developing 2–6 weeks after the peak of the COVID-19 epidemic (15). Another studys (4, 5) also reported that the peak onset time of MIS-C is approximately 4–5 weeks behind the regional peak onset of COVID-19, and the delayed onset of symptoms coincides with the time of the acquired immune response, indicating that MIS-C may be a new type of COVID-19 abnormal immune response after infection. The RT-PCR test results for SARS-CoV-2 in some children with MIS-C were positive, and SARS-CoV-2-related IgM and IgG antibodies could also be detected, indicating that MIS-C and SARS-CoV-2 infection are closely related (6, 16). A study also discovered that a cytokine storm occurred in patients with severe COVID-19, accompanied by severe immune function damage (7, 17). Children with MIS-C have markedly increased levels of inflammatory factors such as IL-4, IL-6, IL-12/IL-23, IL-1β, TNF-β, and ferritin (8, 18). In addition, the timing of the interferon (IFN) response to SARS-CoV-2 infection can vary with viral load and genetic differences in host response. When viral load is low, IFN responses are engaged and contribute to viral clearance, resulting in mild infection. When viral load is high and/or genetic factors slow antiviral responses, virus replication can delay the IFN response, and cytokine storm can result before adaptive responses clear the virus, resulting in severe disease including multisystem inflammatory syndrome in children (MIS-C) (9, 19). The pathogenesis of MIS-C may also be related to damage to endothelial damage induced by SARS-CoV-2. Studies have shown that SARS-CoV-2 can invade endothelial cells, leading to endothelial cell damage and thrombosis. Multiple organ damage may be associated with endothelial damage caused by viral infections (10, 20, 21).

### Diagnosis

At present, there are 3 preliminary diagnostic criteria for MIS-C in the world. (1) Royal College of Pediatrics and Child Health (UK) preliminary diagnostic criteria (13, 22): pediatric age group, persistent fever, and evidence of single or multiorgan dysfunction (shock, cardiac, respiratory, renal, gastrointestinal, or neurological disorder) with additional features, which may include children fulfilling full or partial criteria for Kawasaki disease. SARS-CoV-2 PCR testing may be either positive or negative. (2) Centers for Disease Control and Prevention (United States) preliminary diagnostic criteria (14, 23): (1) age < 21 years; (2) fever for at least 24 h≥38.0 °C; (3) serious illness leading to hospitalization; (4) 2 or more organ systems affected (e.g., cardiac, renal, respiratory, hematologic, gastrointestinal, dermatologic, and neurological); (5) positive for current or recent SARS-CoV-2 infection by RT-PCR, serology, or antigen test; or COVID-19 exposure within the 4 weeks prior to the onset of symptoms. (3) World Health Organization (15, 24): (1) age < 19 years old; (2) persistent fever for more than 3 days; (3) at least two concomitant symptoms; (i) rash or bilateral non-purulent conjunctivitis or mucocutaneous signs (oral, hands, or feet); (ii) hypotension or shock; (iii) features of myocardial dysfunction, pericarditis, valvulitis, or coronary abnormalities (including echocardiography findings or elevated troponin/NT-pro-BNP), (iv) evidence of coagulopathy (by PT, APTT, elevated d-dimers), (v) acute gastrointestinal problems (diarrhea, vomiting, or abdominal pain); (4) elevated inflammatory markers, such as erythrocyte sedimentation rate (ESR), C-reactive protein (CRP) or procalcitonin (PCT), serum ferritin, etc; (5) exclusion of inflammation caused by other pathogenic microorganisms; (6) evidence of COVID-19 infection.

### Differential diagnosis

Overlapping features between MIS-C and KD make diagnosis challenging. Some patients with MIS-C have features resembling KD, a vasculitis of medium-sized vessels, particularly coronary arteries. Like KD, MIS-C often presents with fever, conjunctivitis, rash, extremity changes, mucocutaneous findings, and adenopathy, but several epidemiological differences suggest distinct pathologic mechanisms. MIS-C is a severe inflammatory syndrome similar to Kawasaki disease (KD), nevertheless, differs from KD in several clinical features. Toxic shock and vasoplegic shock are more common in patients with MIS-C but are unusual in classic KD. Once MIS-C occurs, it often affects multiple systems, most of which are critically ill and can progress and worsen rapidly in a short time. Despite the overlapping features of MIS-C and Kawasaki disease, they appear to have distinct inflammatory pathways.

Age Patients with MIS-C typically affect older children and adolescents, with a median age at presentation of at least 8 years(range = 2 weeks−20 years), whereas KD typically affects infants and young children, with a median age of 2 years (12, 25–28).

Ethnicity Despite high COVID-19 caseloads in Asia, such as China, Korea, and Japan–countries with the highest worldwide KD incidences, MIS-C has been reported rarely in Asia (29). The main affected population is in In several series, MIS-C patients may be seen more often in children of Europe, United States, and African ethnicity (25). It is suggested that the occurrence of MIS-C may be related to ethnicity susceptibility.

Clinical features MIS-C reports, characterized predominantly by shock, cardiac dysfunction, abdominal pain, and markedly elevated inflammatory markers, and almost all had positive SARS-CoV-2 test results, and relatively few classic KD criteria when compared with children with KD. Approximately two-thirds did not have preexisting underlying medical conditions before MIS-C onset (2, 26–28).

Cardiovascular features Although both KD and MIS-C can have cardiovascular involvement, the nature of this involvement appears to differ between the two syndromes. Cardiac features of MIS-C most dramatically show moderate to very severe myocardial involvement (manifested by imaging and strikingly high NT-pro-BNP and troponin levels), much greater than associated with KD or KD shock syndrome, As distinct from KD, left ventricular dysfunction is the predominant cardiac feature in patients with MIS-C and the proportion of cardiac dysfunction in MIS-C was considerably higher. In KD, the cardiac hallmark, of course, is coronary artery abnormalities (2, 26–28).

Laboratory features Laboratory features of MIS-C are also quite distinct from those in KD, with greater resemblance to those of MAS (elevated ferritin, D-dimers, triglycerides) and the cytokine storm of TSS. When comparing laboratory testing, the inflammatory markers are more elevated in MIS-C when compared with KD [most notably c- reactive protein (CRP), ferritin, and D-dimer] and the absolute lymphocyte count and platelets tend to be lower in MIS-C when compared to KD (28).

Inflammatory pathways KD and MIS-C have distinct inflammatory pathways. The inflammatory response in MIS-C differs from the cytokine storm of severe acute COVID-19, shares several features with KD, but also differs from this condition with respect to T cell subsets, interleukin (IL)-17A, and biomarkers associated with arterial damage. Autoantibody profiling suggests multiple autoantibodies that could be involved in the pathogenesis of MIS-C. The inflammation of Kawasaki disease is thought to be mediated by autoantibodies following infection in a genetically predisposed host (2, 14).

Etiology KD etiological studies have confirmed that its pathogenesis is related to viral, bacterial, or mycoplasma infection, while MIS-C is primarily found in areas where SARS-CoV-2 is widespread, along with COVID-19 relevant evidence or relevant epidemiological history.

### Treatment

Given that MIS-C has symptoms similar to KD, treatment regimens have been extrapolated from the guidelines for the management of patients with KD. All children meeting the WHO case definition criteria for MIS-C should be monitored in the hospital with possible admission to the PICU and early involvement of a multidisciplinary team. Rapid and aggressive treatment options should be considered according to the evolution of the disease.

Currently, the treatment of MIS-C refers to the American College of Rheumatology clinical guidelines for SARS-CoV-2-associated multisystem inflammatory syndrome in children (30–32). Current practices and published guidelines for the treatment of MIS-C support the use of intravenous immunoglobulin (IVIG) and/or corticosteroids as a first-line cornerstone of therapy (33). In addition to antithrombotic therapy and second-line treatment with different immunomodulatory drugs (e.g., tumor necrosis factor inhibitor, interleukin-1 inhibitor, or interleukin-6 inhibitor), other supportive therapeutic agents are concomitantly used (34, 35). Consensus guidelines support the use of high-dose IVIG for MIS-C patients, administered in a single dose at 2 g/kg based on ideal body weight (max 100 g), which can sometimes be used as needed before full diagnostic evaluation is completed. Glucocorticoids are used in low doses as adjunctive therapy in patients with the moderate-to-severe disease or high doses as intensification therapy in patients with the refractory disease (7, 36). Currently, the treatments of MIS-C are based on treatment strategies in KD or COVID-19, and it is reported some new treatment strategies are effective, such as treatments related to pathogenesis. Anti-cytokine therapy such as anakinra, tocilizumab, or infliximab has been used in cases refractory to first-line treatments (37). An accurate assessment is the first step of antithrombotic therapy, Aspirin is used as a thromboprophylaxis in MIS-C, low-dose aspirin (3–5 mg/kg/day, maximum 81 mg/day). In addition to aspirin, the concomitant use of anticoagulation, such as low molecular weight heparin.

## Conclusions

In conclusion, MIS-C is a hyper-inflammatory syndrome affecting multiple organs and is triggered by SARS-CoV-2 infection, high titres of anti-SARS-CoV-2 antibodies are seen in these patients. MIS-C develops following SARS-CoV-2 infection, and presumably, those adaptive immune mechanisms have a major role to play in the pathogenesis of this condition. Although clinical manifestations of MIS-C and KD may be overlapping, some of the clinical manifestations of MIS-C mimic KD shock syndrome, these appear to be two distinct clinical entities, it is the cardiovascular manifestations that are most prominent. MIS-C cases have now been reported from several countries the world over, while it has been rarely reported in China, which should be closely related to China's scientific epidemic prevention and control measures, Other possible causes need further study. Current treatment guidelines recommend the use of intravenous immunoglobulin (IVIG) and/or corticosteroids as the first-line cornerstone of therapy for MIS-C, However, the new therapy will be used as in cases refractory to first-line treatments need to be further studied. COVID-19 is still a pandemic around the world, and the pathogenesis, diagnostic standard, and treatment of MIS-C require further research and exploration. More attention should be paid to children with COVID-19-related evidence or epidemiological history.

## Data availability statement

The original contributions presented in the study are included in the article/supplementary material, further inquiries can be directed to the corresponding author/s.

## Ethics statement

Written informed consent was obtained from the individual(s), and minor(s)' legal guardian/next of kin, for the publication of any potentially identifiable images or data included in this article.

## Author contributions

Guarantor of integrity of the entire study: W-yW and Y-jW. Study concepts: Y-jW. Study design: C-xA. Definition of intellectual content: HL. Literature research: BY. Data acquisition: W-yW. Data analysis: Q-jZ. Statistical analysis: S-yW and W-yL. Manuscript preparation: W-yW. Manuscript editing: W-yW, Y-jW, and HL. Manuscript review: Y-jW and HL. All authors contributed to the article and approved the submitted version.

## Conflict of interest

The authors declare that the research was conducted in the absence of any commercial or financial relationships that could be construed as a potential conflict of interest.

## Publisher's note

All claims expressed in this article are solely those of the authors and do not necessarily represent those of their affiliated organizations, or those of the publisher, the editors and the reviewers. Any product that may be evaluated in this article, or claim that may be made by its manufacturer, is not guaranteed or endorsed by the publisher.
